# Glutamate transporter splice variant expression in an enriched pyramidal cell population in schizophrenia

**DOI:** 10.1038/tp.2015.74

**Published:** 2015-06-09

**Authors:** S M O'Donovan, K Hasselfeld, D Bauer, M Simmons, P Roussos, V Haroutunian, J H Meador-Woodruff, R E McCullumsmith

**Affiliations:** 1 Department of Psychiatry and Behavioral Neuroscience, University of Cincinnati, Cincinnati, OH, USA; 2 Department of Neuroscience, Wellesley College, Wellesley, MA, USA; 3 Department of Psychiatry, University of Alabama, Birmingham, AL, USA; 4 Department of Psychiatry, Department of Genetics and Genomic Sciences, and Institute for Genomics and Multiscale Biology, Icahn School of Medicine at Mount Sinai, New York, NY, USA; 5 James J. Peters VA Medical Center, Mental Illness Research Education and Clinical Center, Bronx, NY, USA; 6 Department of Psychiatry and Neuroscience, Icahn School of Medicine at Mount Sinai, New York, NY, USA

## Abstract

Dysregulation of the glutamate transporters EAAT1 and EAAT2 and their isoforms have been implicated in schizophrenia. EAAT1 and EAAT2 expression has been studied in different brain regions but the prevalence of astrocytic glutamate transporter expression masks the more subtle changes in excitatory amino acid transporters (EAATs) isoforms in neurons in the cortex. Using laser capture microdissection, pyramidal neurons were cut from the anterior cingulate cortex of postmortem schizophrenia (*n*=20) and control (*n*=20) subjects. The messenger RNA (mRNA) levels of EAAT1, EAAT2 and the splice variants EAAT1 exon9skipping, EAAT2 exon9skipping and EAAT2b were analyzed by real time PCR (RT-PCR) in an enriched population of neurons. Region-level expression of these transcripts was measured in postmortem schizophrenia (*n*=25) and controls (*n*=25). The relationship between selected EAAT polymorphisms and EAAT splice variant expression was also explored. Anterior cingulate cortex pyramidal cell expression of EAAT2b mRNA was increased (*P*<0.001; 67%) in schizophrenia subjects compared with controls. There was no significant change in other EAAT variants. EAAT2 exon9skipping mRNA was increased (*P*<0.05; 38%) at region level in the anterior cingulate cortex with no significant change in other EAAT variants at region level. EAAT2 single-nucleotide polymorphisms were significantly associated with changes in EAAT2 isoform expression. Haloperidol decanoate-treated animals, acting as controls for possible antipsychotic effects, did not have significantly altered neuronal EAAT2b mRNA levels. The novel finding that EAAT2b levels are increased in populations of anterior cingulate cortex pyramidal cells further demonstrates a role for neuronal glutamate transporter splice variant expression in schizophrenia.

## Introduction

Excitatory amino acid transporters (EAATs) mediate clearance of glutamate levels in the central nervous system preventing pathological accumulation of glutamate in the synapse.^1^ The EAAT family consists of five transporters (EAAT1–5) with different regional and cellular localizations. Of particular interest in schizophrenia are EAAT1 and EAAT2 (ref. 2) and their well-studied rodent analogs, GLAST^3^ and GLT1,^4^ respectively. EAAT2 is the primary glutamate transporter in the brain and is responsible for clearing over 90% of glutamate from the synapse in most regions.^5, 6, 7^

In schizophrenia, expression of EAAT1 and EAAT2 and their interacting proteins are altered in both the thalamus and cortex.^8, 9, 10^ EAAT1 and EAAT2 expression is generally found in glial cells.^11^ However, recent microscopy studies have expanded our understanding of the cellular localization of EAAT1 and EAAT2 in human brain. Electron microscopy studies have confirmed EAAT1 and EAAT2 immunoreactivity in neurons in the cortex of normal postmortem brain.^12^ Although glial expression of EAAT1 and EAAT2 is well established, neuronal expression of EAAT1 and EAAT2 in schizophrenia has yet to be studied.

Multiple isoforms of EAAT1 and EAAT2 are expressed in the cortex with differing levels of functionality.^13, 14, 15, 16^ There are several exon-skipping splice variants including EAAT2 exon9skipping (exon9 deletion), which lacks parts of the glutamate translocation site. The EAAT1 exon9skipping variant lacks the exon homologous to that of EAAT2 exon9skipping.^17^ EAAT2b is a 3′ variant that contains an alternative C-termini and was thought to be primarily expressed in astrocytes.^18^ Glutamate transporter splice variant expression has been implicated in various neuropsychiatric conditions including amyotrophic lateral sclerosis, Alzheimer's disease and hypoxia.^19, 20^ EAAT1 and EAAT2 splice variant expression has been measured at the region level only in schizophrenia.^21^ In this study, we measured cell-level changes in the messenger RNA (mRNA) expression of EAAT1 and EAAT2 isoforms. We hypothesize that cell-level changes in expression of glutamate transporters occur in pyramidal neurons in schizophrenia. These subtle alterations in neuronal EAAT expression, particularly in the splice variants, may have a little understood role in the pathophysiology of this disorder.

## Materials and methods

### Subjects

Subjects from the Mount Sinai Medical Center Schizophrenia Brain Bank were studied (Table 1), including 25 individuals diagnosed with schizophrenia and 25 comparison subjects for region-level comparisons and 20 individuals diagnosed with schizophrenia and 20 comparison subjects for cell-level studies. Nine schizophrenia and 10 control subjects were common to both region-level and cell-level studies. Subjects were matched for age, postmortem interval (PMI) and pH (Table 1). The medical records of the subjects designated as controls were examined using a formal masked medical chart review instrument as well as in-person interviews with the subjects and/or their caregivers. The subjects were evaluated for NINCDS-AIREN criteria for a diagnosis of vascular dementia; NINCDS, DSMIV and CERAD diagnosis of dementia; consensus criteria for a clinical diagnosis of probable or possible diffuse Lewy body disease; UPDRS for Parkinson's disease; clinical criteria for diagnosis of frontotemporal dementia; medical history of psychiatric disease; history of drug or alcohol abuse; and other tests of cognitive function including the mini-mental state examination and clinical dementia rating. In addition, each brain tissue specimen was examined neuropathologically using systematized macro- and microscopic evaluation using CERAD guidelines. As the patients in our cohort were elderly at the time of death, many of the subjects have the cognitive impairment associated with aged subjects with schizophrenia.^22, 23, 24, 25^ All samples were derived from the left side of the brain. Subjects with schizophrenia were diagnosed with this illness for at least 30 years. The brain banking procedures were approved by the Mount Sinai School of Medicine Institutional Review Board.

Brains were obtained after autopsy and one hemisphere was prepared. For region-level studies, tissue was cut coronally into ~0.8–1 cm^3^ slabs and flash frozen. Gray matter was dissected from anterior cingulate cortex (ACC) and dorsolateral prefrontal cortex. ACC was dissected at the level of the genu of the corpus callosum, from the dorsal surface of the corpus callosum extending 12–15 mm dorsally and extending 12–15 mm laterally from the midline. Dorsolateral prefrontal cortex was dissected corresponding to Brodmann area 46 measuring ~1.5 cm along the cortical surface. Approximately 1 cm^3^ of frozen tissue was pulverized in liquid nitrogen.

For cell-level studies, brains were cut coronally into 10 mm slabs and frozen until further dissection. Fifteen micrometer sections were mounted on 1 × 3 inch penfoil polymer or superfrost plus glass slides. After sectioning, the ACC was grossly dissected from the remaining tissue blocks. The last section from the tissue block for each case was Nissl stained and used as a guide for dissections. The ACC was identified using detailed atlases and (previously published) *in situ* hybridization studies.^26, 27, 28, 29, 30, 31, 32^

For region-level studies, tissue was homogenized in Buffer RLT Plus (AllPrep DNA/RNA Mini Kit (Qiagen, Limburg, The Netherlands)) by passing it 10 times through a 20-guage needle and RNA was extracted with the AllPrep DNA/RNA Mini Kit (Qiagen). RNA concentration was determined using UV spectrophotometry at 260 nm. Equivalent amounts of RNA (1 g) were treated with DNase I at 37 °C for 30 min (1 unit DNase per gram RNA; Promega, Madison, WI, USA), DNase was inactivated at 65 °C for 15 min, and RNA was reverse transcribed using the High-Capacity cDNA Archive Kit (Applied Biosystems, Foster City, CA, USA).

To conduct cell-level studies of EAAT mRNA expression, an enriched population of pyramidal cells (500 cells per subject) were identified and cut from the ACC by laser microdissection (Supplementary Figure 1) using the Veritas Microdissection instrument and CapSure Macro LCM caps (Life Technologies, formerly Arcturus, Mountain View, CA, USA). Frozen tissue sections on SuperfrostPlus glass (25 mm × 75 mm) slides were thawed at room temperature and fixed with PALM Liquid CoverGlass N (P.A.L.M. Microlaser Technologies, Bernried, Germany) and allowed to air dry. Tissue sections were rehydrated with distilled H_2_0 and then underwent rapid Nissl staining with an RNAse-free cresyl violet solution (1% cresyl violet, 1% glacial acetic acid, pH 4.0). Slides were then dehydrated through serial ethanol washes and soaked in xylene for 10 min. Microdissection was performed under the × 20 objective lens with laser settings ranging from 70 to 100 mW in power, and 2000 to 3000 μs in duration. Separate caps were used for each subject and each cell population. Following cell capture, each cap was incubated with 50 μl of PicoPure RNA extraction buffer (Life Technologies, Carlsbad, CA, USA) in a 0.5 ml microcentrifuge tube (Applied BioSystems) for 30 min at 42 °C. Samples were then centrifuged for 2 min at 800 *g* and stored at −80 °C.

RNA was isolated from the laser capture microdissected neurons using the PicoPure RNA isolation kit (Molecular Devices, Sunnyvale, CA, USA) according to the manufacturer's protocol. The complementary DNA (cDNA) was synthesized using a High-Capacity cDNA Reverse Transcription Kit (Applied Biosystems) using 8 μl of total RNA.

The SYBR-Green primer pairs (Supplementary Table 1) were pooled and diluted with TE buffer (10 mM Tris-Cl, 1 mM EDTA, pH 8.0) to a final concentration of 0.2 × and were combined with SYBR-Green Master Mix (Applied Biosystems) and cDNA for the preamplification PCR reaction. Custom-made assays were tested for specificity and resulted in a single band of expected size, which was confirmed with excision and sequencing (data not shown). The PCR cycles were: 1 cycle of denaturing at 95 °C for 10 min, then 14 cycles of denaturing at 95 °C for 14 s and annealing at 60 °C for 4 min. Pre-amplified samples were diluted 1:3 with TE buffer and stored at 20 °C until used in real-time PCR (RT-PCR) assays.

### RT-PCR assays

SYBR-Green Quantitative RT-PCR reactions were performed in duplicate using 96-well optical reaction plates (Stratagene, LaJolla, CA, USA) on a Stratagene detection system. For each reaction, 3 μl of cDNA (1:3 diluted) was placed in a 20 μl reaction containing 10 μl of SYBR-Green PCR Master Mix (Applied Biosystems) and 10 pmol of each primer (Invitrogen, Carlsbad, CA, USA). The primers used are listed in Supplementary Table 1. Reactions were performed with an initial ramp time of 3 min at 95 °C, and 50–60 subsequent cycles of 15 s at 95 °C and 1 min at the annealing temperature. The annealing temperature was 63 °C for EAAT2 exon9skipping and was 59 °C for all other primer sets. In all, 60 cycles were used for EAAT2b and EAAT2 exon9skipping, and 50 cycles were used for all other primer sets. For negative controls for the quantitative RT-PCR reactions, cDNA was omitted. Relative concentrations of the transcripts of interest were calculated with comparison to a standard curve made with dilutions of cDNA from a pooled sampling of all the subjects. Values for the transcripts of interest were normalized to the geometric mean of 18 s, actin, GAPDH and cyclophilin A values for the same samples.

### RNA integrity analysis

Twenty micrometer tissue sections were scraped from glass slides using a sterile scalpel. Total RNA was isolated using the Trizol reagent and protocol. RNA samples were analyzed using protocols and supplies described in Agilent RNA 6000 Nano Kit guide (Cat. #5067-1511). RNA integrity (RIN) values were generated with the Agilent 2100 bioanalyzer (software ver. B.02.07.SI532; Agilent Technologies, Santa Clara, CA, USA).

### Animal treatments

Rodent studies were performed in accordance with the IACUC guidelines at the University of Alabama at Birmingham. Adult male Sprague–Dawley rats (250 g) were housed in pairs and maintained on a 12-h light/dark cycle. Rats received 28.5 mg kg^−1^ haloperidol decanoate or vehicle (sesame oil) by intramuscular injection every 3 weeks for 9 months. Brain tissue was dissected and stored at −80 °C.

### Animal RT-PCR assays

EAAT splice variant mRNA expression changes were compared in haloperidol-treated (*n*=10) and control (*n*=10) rats at region level and cell level. For region-level studies, the frontal cortex of each rodent was sectioned (14 μm sections) on a cryostat (Cryostar NX70, ThermoScientific, Waltham, MA, USA) following removal of the olfactory bulbs and frontal pole. Three sections from each rodent were processed. For cell-level experiments, 500 pyramidal cells were cut from Nissl-stained sections from the rat frontal cortex (14 μm) by laser capture microdissection, as described above. For both region- and cell-level experiments, RNA was isolated using the Arcturus PicoPure RNA isolation kit (Applied Biosystems) according to the manufacturer's protocol. cDNA was then reverse transcribed and analyzed as described above using Sybr-Green quantitative RT-PCR. The primers used are listed in Supplementary Table 2.

### Single-nucleotide polymorphism study

DNA was extracted with the ZR Genomic DNA-Tissue MiniPrep Kit (Zymo Research, Irvine, CA, USA) from 50 mg of human STG brain tissue. High-throughput, genome-wide genotyping was performed, using the Affymetrix 6.0 technology, at the Center for Applied Genomics at CHOP. DNA was processed according to the instructions provided in the Affymetrix Genome-Wide Human SNP Nsp/Sty 6.0 Assay Manual. Initial analysis of the array to obtain intensity data was performed using Affymetrix Power Tools. Quality control measures were calculated on the Affymetrix 6.0 data based on statistical distributions to exclude poor-quality DNA samples. Samples were excluded (1) if single-nucleotide polymorphism (SNP) call rate was <90% (2) patterns of SNP genotypes revealed duplicates or if the heterozygosity was >3 s.d. above the mean; or (3) if patterns of SNP genotypes revealed cryptic relatedness determined by pairwise identity-by-state analysis.

### Statistical analysis

All data sets were analyzed for normal distribution. Outliers >3 s.d. from the mean were excluded. Correlation analysis was performed to determine associations between transcript expression and age, PMI and RIN value. One-way analysis of covariance was performed if significant correlations were found. If no correlations were present, data were analyzed with Student's *t*-test. The significance level was *P*<0.05 for all the statistical tests. Secondary analyses were performed to assess the effects of sex and medication on the dependent measures. To test for possible medication effects, patients with schizophrenia off antipsychotic medication for at least 6 weeks before death were compared with patients on antipsychotic medication within 6 weeks of death in a *post hoc* analysis. All data sets were analyzed with Statistica (Statsoft, Tulsa, OK, USA).

Rodent data were analyzed for normal distribution. Outliers >3 s.d. from the mean were excluded. Data were log transformed and analyzed by Student's *t*-test and the significance level was *P*<0.05 for all the statistical tests. Data were analyzed with Graphpad Prism v6.04 (GraphPad Software, San Diego, CA, USA).

### SNP statistical analysis

Genotype frequency was observed by direct counting. Hardy–Weinberg equilibrium was examined for all polymorphisms using Haploview software.^33^ Mann–Whitney *U*-test was applied to examine log-transformed splice variant mRNA expression in subjects homozygous for the major allele and subjects who were carriers (homozygous and heterozygous) of the minor allele. Data were analyzed with Graphpad Prism v6.04 (GraphPad Software).

## Results

### Cell-level schizophrenia studies

The expression levels of the glutamate transporters, EAAT1 and EAAT2, and the splice variants, EAAT1 exon9skipping, EAAT2 exon9skipping and EAAT2b, were measured in an enriched population of ACC pyramidal cells cut by laser capture microdissection to ensure greater cell specificity of the assays.

**Figure 1:**
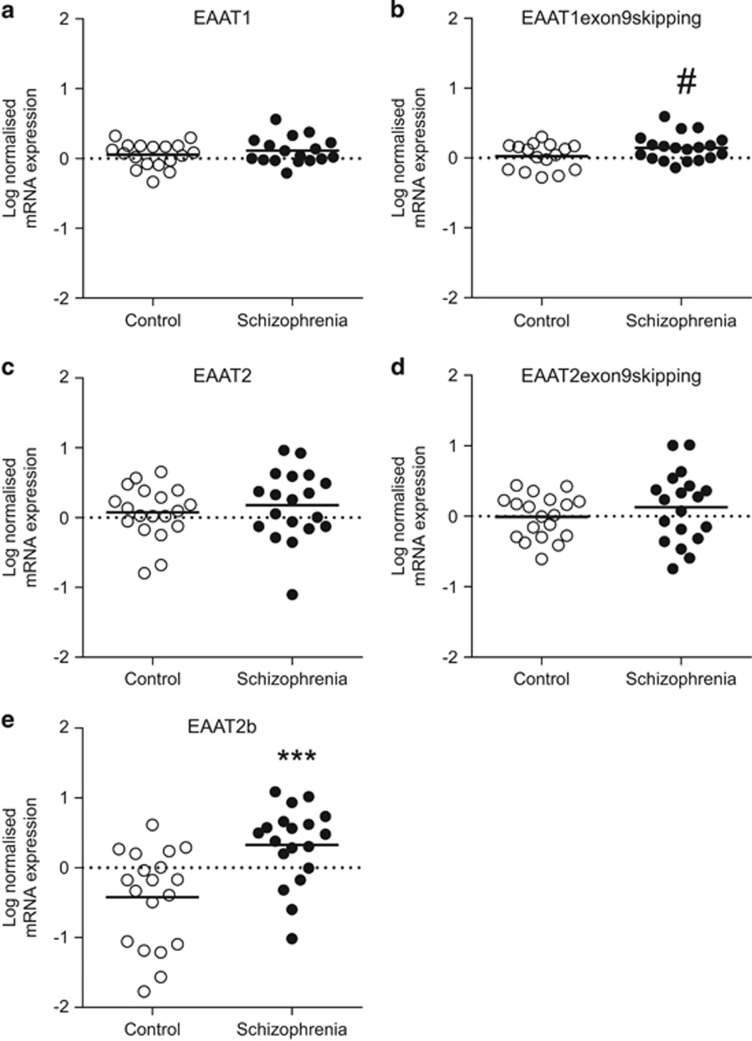
Cell-level ACC log-normalized mRNA expression of the EAAT splice variants EAAT1 (**a**), EAAT1 exon9skipping (**b**), EAAT2 (**c**), EAAT2 exon9skipping (**d**) and EAAT2b (**e**). Following Student's *t*-test analysis, there was a significant difference in EAAT2b splice variant expression in schizophrenia subjects compared with controls in ACC pyramidal cells (*P*<0.001). There was a trend toward an increase in EAAT1 exon9skipping mRNA expression in schizophrenia subjects (*P*<0.07). Data are expressed as mean±s.d., *n*=16–19 per group. ***P<0.001, ^#^*P*<0.07. ACC, anterior cingulate cortex; EAAT, excitatory amino acid transporter; mRNA, messenger RNA.

In the ACC cell-level study, there was no significant association between expression of the target mRNAs EAAT1, EAAT2, EAAT1 exon9skipping, EAAT2 exon9skipping, EAAT2b and age, sex or RIN values. Student's *t*-test revealed a significant increase (67%) in EAAT2b mRNA expression (F_(1,36)_=13.762, *P*=0.0007) in an enriched population of ACC pyramidal neurons in schizophrenia compared with controls (Figure 1e). There was no significant effect of sex or medication on expression. There was a trend toward an increase (10%) in EAAT1 exon9skipping mRNA expression (F_(1,33)_=3.565,*P*=0.068; Figure 1b). There was no sex or medication effect for EAAT1 exon9skipping. There were no significant changes in mRNA expression for EAAT1, EAAT2 or EAAT2 exon9skipping (Figures 1a, c and d).

### Cell-level animal studies

**Figure 2:**
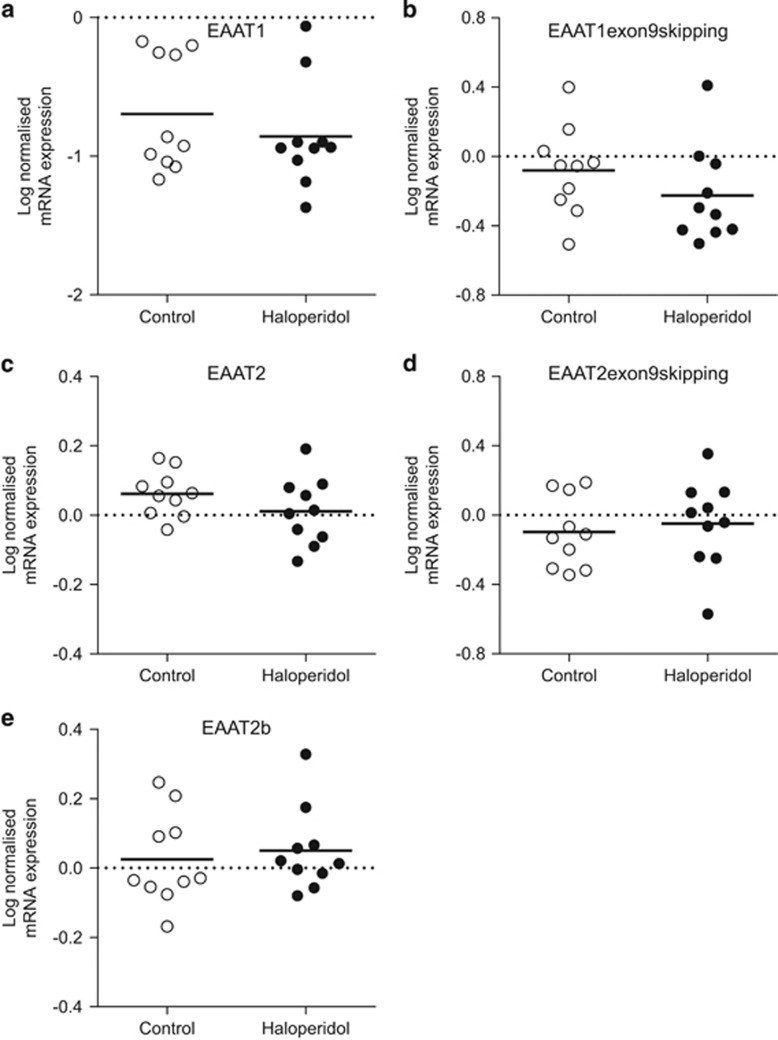
Rat haloperidol-treated cell-level log-normalized mRNA expression of the EAAT splice variants EAAT1 (**a**), EAAT1 exon9skipping (**b**), EAAT2 (**c**), EAAT2 exon9skipping (**d**) and EAAT2b (**e**). Following Student's *t*-test analysis, there was no significant difference in splice variant expression in pyramidal cells from the frontal cortex of haloperidol-treated animals compared to controls. Data are expressed as mean±s.d., *n*=9–10 per group. EAAT, excitatory amino acid transporter; mRNA, messenger RNA.

Student's *t*-test revealed no significant effect of haloperidol administration on EAAT1, EAAT1 exon9skipping, EAAT2, EAAT2 exon9skipping or EAAT2b splice variant mRNA expression in pyramidal cells cut from the rat frontal cortex (Figure 2).

### Region-level schizophrenia studies

In the dorsolateral prefrontal cortex region-level study, there were no significant associations between expression of the target mRNAs, EAAT1, EAAT2, EAAT1 exon9skipping, EAAT2 exon9skipping, EAAT2b and age, PMI or RIN values. Student's *t*-test revealed no significant differences in mRNA expression between control and schizophrenia subjects (Supplementary Figures 2A–E).

**Figure 3:**
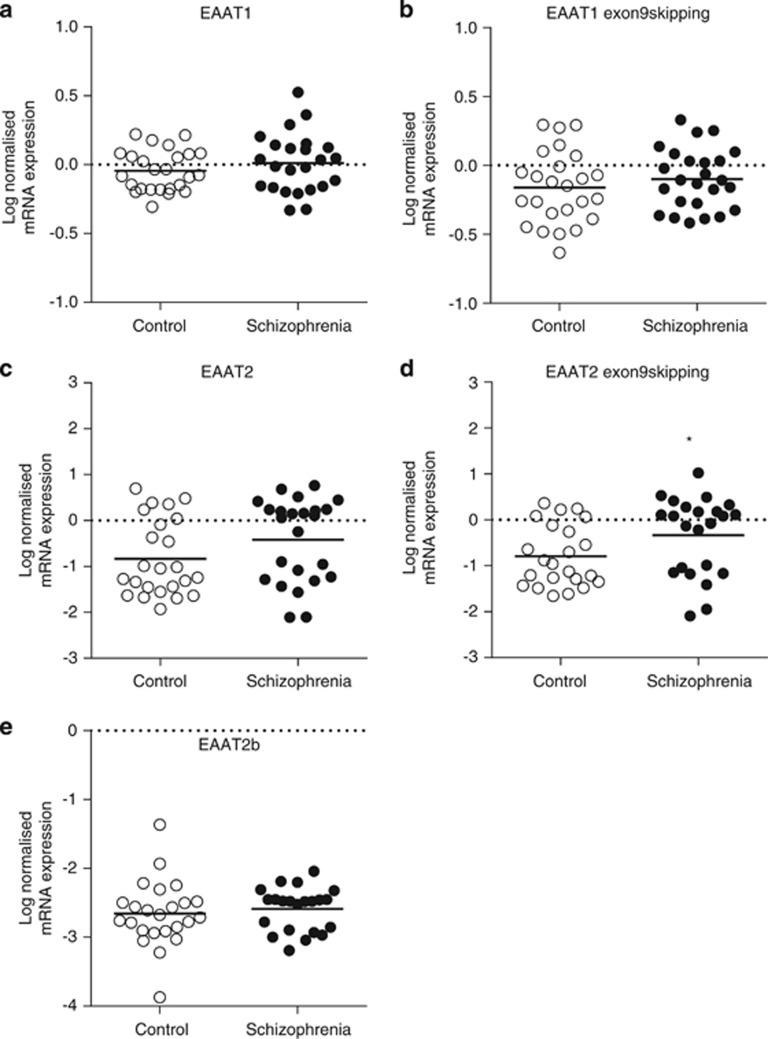
Region-level ACC log-normalized mRNA expression of the EAAT splice variants EAAT1 (**a**), EAAT1 exon9skipping (**b**), EAAT2 (**c**), EAAT2 exon9skipping (**d**) and EAAT2b (**e**). Following Student's *t*-test analysis, there was a significant difference in EAAT2 exon9skipping splice variant expression in schizophrenia subjects compared with controls in the ACC at region level (*P*<0.05). Data are expressed as mean±s.d., *n*=22–25 per group. *P<0.05. ACC, anterior cingulate cortex; EAAT, excitatory amino acid transporter; mRNA, messenger RNA.

In the ACC region-level study, there were no significant associations between expressions of the target mRNAs EAAT1, EAAT2, EAAT1 exon9skipping, EAAT2 exon9skipping, EAAT2b and age, PMI or RIN values. Student's *t*-test revealed a significant increase in EAAT2 exon9skipping mRNA expression in schizophrenia compared with controls (F_(1,44)_=4.21, *P*=0.046, 38% increase; Figure 3d). There was no significant effect of sex or medication on EAAT2 exon9skipping expression. There was no significant change in mRNA expression in EAAT1, EAAT2, EAAT1 exon9skipping or EAAT2b (Figures 3a–c, e).

### Region-level animal studies

Student's *t*-test revealed a significant increase in EAAT2b mRNA expression (*P*<0.001, 31% increase) in the frontal cortex in rats administered haloperidol decanoate for 9 months (Supplementary Figure 3E). There was no significant effect of haloperidol decanoate treatment on mRNA expression of EAAT1, EAAT1 exon9skipping, EAAT2 or EAAT2 exon9skipping splice variants (Supplementary Figures 3A–D).

### SNP results

**Figure 4:**
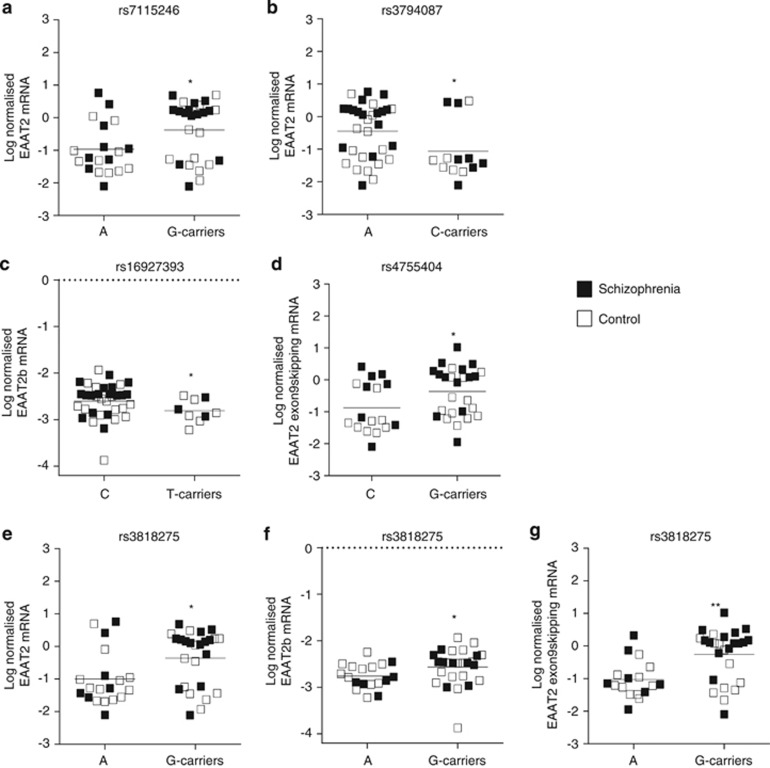
There were significant associations between EAAT2 SNPs and the log-normalized mRNA expression of EAAT2 splice variants in region-level ACC following Mann–Whitney *U* analysis. EAAT2 mRNA expression was significantly altered (*P*<0.05) in SNP rs7115246 (**a**) and rs3794087 (**b**) subjects with the A/A polymorphism compared with G-carriers or C-carriers, respectively. EAAT2b mRNA expression was significantly altered (*P*<0.05) in SNP rs16927393 (**c**) subjects with the C/C polymorphism compared with T-carriers. EAAT2 exon9skipping mRNA expression was significantly altered (*P*<0.05) in SNP rs4755404 (**d**) subjects with the C/C polymorphism compared with G-carriers. EAAT2, EAAT2b and EAAT2 exon9skipping mRNA expression was significantly altered (*P*<0.05–*P*<0.01) in SNP rs3818275 (**e**–**g**) subjects with the A/A polymorphism compared with G-carriers. Filled squares represent schizophrenia subjects, open squares represent control subjects. Data are expressed as mean±s.d., *n*=9–32 per group. *P<0.05, **P<0.01. EAAT, excitatory amino acid transporter; EAAT2ex9, EAAT2 exon9 skipping splice variant; mRNA, messenger RNA; SNP, single-nucleotide polymorphism.

All EAAT2 and EAAT1 polymorphisms were in Hardy–Weinberg equilibrium. We found several significant associations between SNPs in the EAAT2 (SLC1A2) gene and EAAT2 splice variant mRNA expression (Supplementary Table 3). SNP rs7115246 G-allele carriers had significantly increased (*P*<0.05) EAAT2 mRNA expression compared with homozygous A-allele carriers (Figure 4a). EAAT2 mRNA was significantly decreased (*P*<0.05) in SNP rs3794087 C-carriers compared with homozygous A-allele carriers (Figure 4b). EAAT2b mRNA was significantly decreased (*P*<0.05) in SNP rs16927393 T-carriers compared with homozygous C-allele carriers (Figure 4c). EAAT2 exon9skipping mRNA was significantly increased (*P*<0.05) in SNP rs4755404 G-carriers compared with homozygous C-allele carriers (Figure 4d). SNP rs3818275 was associated with increases in EAAT2 (*P*<0.05), EAAT2b (*P*<0.05) and EAAT2 exon9skipping (*P*<0.01) mRNA levels in G-carriers compared with homozygous A-allele carriers (Figure 4e–g). There were no significant effects of the EAAT1 (SLC1A3) SNPs on EAAT1 or EAAT1 exon9skipping mRNA expression (Supplementary Table 3).

## Discussion

Cell-level analysis of EAAT mRNA expression in a population of ACC pyramidal cells found a significant increase (*P*<0.001, 67% increase on control levels) in neuronal EAAT2b mRNA in schizophrenia subjects compared with controls. A trend (*P*<0.07, 10% increase on control levels) toward an increase in EAAT1 exon9skipping was also seen in schizophrenia (Figure 1). We did not detect a change in EAAT2b or EAAT1 exon9skipping mRNA in pyramidal neurons from haloperidol decanoate-treated rats (Figure 2), suggesting that the increase in mRNA expression seen in schizophrenia is not a consequence of chronic antipsychotic treatment.

Of particular interest is our finding that EAAT2b mRNA levels are increased in an enriched population of pyramidal cells in the ACC. EAAT2b is structurally identical to EAAT2 except for 11 amino acids at the C terminus which contain a PDZ domain-binding motif, allowing it to interact with scaffolding proteins including postsynaptic density 95.^34, 35^ Unlike other splice variants, including EAAT2 exon9skipping, EAAT2b can transport glutamate when expressed alone, not needing to form a heteromeric complex with EAAT2 to act as an active glutamate transporter.^17^ Consistent with our results, which show an increase of neuronal EAAT2b in schizophrenia subjects compared with controls, glutamate transporter expression in neurons appears to be strongly related to disease state.

In amyotrophic lateral sclerosis, a disease closely associated with EAAT dysfunction, EAAT2b is significantly increased in pyramidal neurons but not in astrocytes.^19^ It has been proposed that neuronal EAAT2b expression in the motor cortex may be increased to compensate for the loss of EAAT2 protein seen in amyotrophic lateral sclerosis, although the levels are not sufficient to counteract the effects of a reduction of astrocytic EAAT2 associated with this disease.^19^ Interestingly, astrocyte overexpression of EAAT2 in primary culture appears to increase neuron survival in response to increased glutamate exposure, whereas overexpression of EAAT2 in neurons renders cells more vulnerable to cell death following glutamate challenge.^36, 37^ In hypoxia, a state where robust GLT1b (rodent EAAT2b analog) expression was observed in damaged but not undamaged cortical layers,^20^ GLT1b was induced in neurons in response to rising glutamate levels and loss of glial glutamate transporters.^38^ Others report induction of the neuronal expression of glutamate transporters in subjects diagnosed with Alzheimer's disease and Lewy body disease but not in control subjects.^39^ In a possible protective role, GLT1b has been proposed to reduce the effects of glutamate receptor activation on the expressing neuron by reducing potential excitotoxic events resulting from increased extracellular glutamate levels.^20^

EAAT2 exon9skipping is expressed in both patients with neurodegenerative disorders and normal controls and may have a role in the normal physiological control of EAAT2 levels.^14, 40, 41^ The EAAT2 exon9skipping variant can form a trimer when expressed alone, however, it must be co-expressed with EAAT2 or EAAT2b to form an active transporter.^1, 17^ In the present study, we found increased levels of EAAT2 exon9skipping mRNA at the region level in the ACC in schizophrenia (Figure 3). We posit that this increase is likely the result of increased expression of this isoform in astrocytes, where EAAT2 is predominately expressed.

Differences between region- and cell-level EAAT2 splice variant mRNA expression highlights the importance of cell-level approaches to study schizophrenia. Similar to our findings, (Supplementary Figure 2), previous studies of dorsolateral prefrontal cortex and primary visual cortex also found no region-level differences in expression of EAAT1, EAAT2 or EAAT2b mRNA.^9, 21^ The greater abundance of glial EAAT2 expression may have masked the changes in EAAT2b levels, which we only detected following cell-level analysis of splice variant expression. Indeed, several authors report changes in EAAT2 protein levels in psychiatric disorders including Alzheimer's disease^42, 43^ and amyotrophic lateral sclerosis,^44^ but with no corresponding changes in mRNA expression. Although several factors could explain why changes in mRNA expression are not detected,^14^ region-level mRNA measures alone may not identify relevant changes in neuronal EAAT2 expression and the contributions of both glial and neuronal expression of EAAT isoforms must be considered.

Overall, our results suggest that ACC pyramidal neurons express alternate isoforms of EAAT2, likely in response to the abnormalities in glial glutamate transporters associated with schizophrenia.^9, 45, 46^ Increases in EAAT2b expression, homomultimers of which form the only functional splice variant transporter,^17^ suggest that neuronal expression of this isoform may be both compensatory and protective in nature. Similar evidence for EAAT2b neuronal expression has been found for other disorders including Alzheimer's disease and hypoxia.^16, 47, 48^

The global effects of neuronal expression of EAAT1 and EAAT2 splice variant expression in disease states are not fully understood. Alternative splicing of EAAT2 was correlated with pathological severity in Alzheimer's disease.^49^ Neurons from Alzheimer's disease subjects containing tangles and abnormal tau protein were in some cases also found to contain EAAT2 protein, implicating neuronal expression of EAAT2 with disease state.^14, 50^ Higher concentrations of the splice variant EAAT2 exon9skipping in neurite and glial processes near amyloid plaques suggested that accumulation of splice variants at sites of neurodegeneration could impair EAAT2 function.^47^ EAAT1 was also identified in Alzheimer's disease in pyramidal cells although it is not yet known whether EAAT1 expression is associated with disease pathology or whether it has a neuroprotective role by supplementing glial function in the reuptake of glutamate from the extracellular space.^49^

It has been well established that EAAT1 and EAAT2 are predominantly expressed in astrocytes.^4, 51^ However, there is now convincing evidence for the neuronal expression of these transporters. EAAT1 and EAAT2 immunoreactivity in neurons has been demonstrated in postmortem brain^12^ where EAAT2 reactivity was detected in dendritic spines, particularly in the postsynaptic density, and in neuronal soma on ribosomes and the rough endoplasmic reticulum.^12^ GLT1 and GLT1b immunoreactivity has been identified in retinal neurons^52^ and in neurons in rat brain culture.^35^ We have now shown that EAAT1 and EAAT2 splice variant mRNA is differentially expressed in an enriched population of pyramidal neurons in the ACC in schizophrenia and controls. Pyramidal cells were identified by their distinctive morphology and specifically targeted and cut by laser capture microdissection, a method effectively used to isolate discrete regions or cells in the study of schizophrenia.^53, 54, 55^ As neurons are typically enveloped by astrocytes, it is possible that EAAT2 mRNA, previously identified by *in situ* hybridization to be present in glial processes,^56^ may be detectable in this neuronal population.^57^ However, we have previously cut small (glial) and large (neuronal) cells from tissue sections and effectively verified the enrichment of these populations using gene expression assays of target cell markers.^54^ Pyramidal cells provide an excellent target for laser capture microdissection studies as they can be easily identified due to their large size and unique morphology. Astrocyte identification provides a greater challenge. However, use of optimized staining and capture protocols can result in greater confidence in cutting an enriched population of these glial cells.^54^

A major consideration in postmortem schizophrenia studies is the potential influence of chronic exposure to antipsychotic treatment. Our human data were examined statistically and found to be nonsignificant for medication effects, although the relatively small sample size of the study may preclude the detection of such effects. To further address this issue, the effects of chronic (9-month) administration of the antipsychotic haloperidol decanoate on rat frontal cortex was studied. At region-level, there was a significant increase in EAAT2b (GLT1b) mRNA in haloperidol decanoate-treated animals (Supplementary Figure 3E). However, in an enriched population of pyramidal cells cut from the frontal cortex, there was no significant effect on any of the splice variants examined, including EAAT2b (GLT1b; Figure 2). Interestingly, in schizophrenia, EAAT2b expression was significantly increased in the ACC in pyramidal neurons, but not in blended, region-level samples. It is possible that astrocytic, but not neuronal EAAT2, expression is affected by haloperidol administration, or alternatively that neuronal EAAT2 mRNA is only impacted in schizophrenia brain. The region-level increase in rat EAAT2b (GLT1b) mRNA is more difficult to interpret but may indicate a previously undetected antipsychotic-induced increase in EAAT2b (GLT1b) in astrocytes where this isoform is also widely expressed.^56^ Further work will be required to explore this scenario.

We also investigated the relationship between SNPs in the SLC1A3 (EAAT1) and SLC1A2 (EAAT2) genes with EAAT1 and EAAT2 splice variant mRNA expression, respectively. Of particular interest are SNPs in the EAAT2 genes, which affect cognitive function in schizophrenia. Cognitive deficits are a fundamental symptom of schizophrenia and can significantly impact patient quality of life.^58^ Several of the SNPs examined in this study were also identified in an association study of EAAT polymorphisms in schizophrenia.^59^

Overall, the role of EAAT2 polymorphisms in schizophrenia have not been examined in depth, with the exception of SNP rs4354668,^58, 60, 61, 62^ which has also been explored in relation to other disorders.^60, 63, 64, 65^ SNP rs4354668 is a T to G polymorphism located 181 base pairs from the EAAT2 gene transcription start site. The G-allele is associated with a 30% reduction in promoter activity compared with the T-allele and is associated with diminished transporter expression.^65, 66^

In schizophrenia subjects, this SNP is associated with a reduced number of categories completed in the Wisconsin Card Sorting Test among G-carriers, an assessment that measures executive function related to abstract thinking.^61^ G-carriers in the study also demonstrated deficits in working memory as assessed by the N-back test. Homozygous G-allele carriers of rs4354668 who are poor performers on the N-back test are also associated with significantly reduced gray matter volume compared with T-carriers.^60^ There is no difference in gray matter volume between G- and T-allele carriers who are good performers on the N-back working memory test. Our study has now identified a panel of EAAT2 polymorphisms that are significantly associated with EAAT2 splice variant mRNA expression changes in schizophrenia (Supplementary Table 3). The small sample size and exploratory nature of this study make it difficult to interpret the relevance of EAAT2 polymorphisms that significantly impact gene expression. However, these results demonstrate that categorizing study subjects on the basis of SNP genotype expression has the potential to provide greater insight into the role of EAAT2 expression changes in schizophrenia. Further studies in a larger sample set will be required to fully determine the associations between genotypes, transporter expression and disease.

### Summary

EAAT1 and EAAT2 splice variant mRNA expression is significantly altered in an enriched population of ACC pyramidal neurons in the schizophrenia postmortem brain. As in other neuropsychiatric disorders,^19^ EAAT2b mRNA expression appears to be particularly affected. The potential role of neuronal expression of EAAT isoforms in schizophrenia has yet to be elucidated. However, it is clear that studies of specific populations of cells will be essential to gain a comprehensive understanding of EAAT expression and function. Coupled with our findings that EAAT2 polymorphisms have a robust effect on splice variant mRNA expression, future studies using these tools will allow us to gain greater insight into the relationship between neuronal EAAT2 regulation and schizophrenia.

## Figures and Tables

**Table 1:** Subject characteristics for splice variant studies

	*Region level*		*Cell level*	
	*Control*	*Schizophrenia*	*Control*	*Schizophrenia*
*N*	25	25	20	20
Sex	12M/13F	16M/9F	9M/11F	13M/7F
Tissue pH	6.6±0.3	6.5±0.2	6.4±0.3	6.4±0.3
PMI (hours)	8.6±7.0	17.1±10.1	10.4±6.1	13.6±6.7
Age (years)	75.8±11.4	75.2±12.9	77.8±10.8	78.6±11.1
On/off Rx	NA	18/7	NA	13/6

Abbreviations: F, female; M, male; NA, not applicable; PMI, postmortem interval; Rx, on/off antipsychotic medication for ⩾6 weeks.

Values are presented as mean±s.d.
