# Supplementary figures and images for: Glutamate transporter splice variant expression in an enriched pyramidal cell population in schizophrenia
Source: Transl Psychiatry. 2015 Jun 9;5(6):e579–. doi: 10.1038/tp.2015.74 (PMC4490284; doi:10.1038/tp.2015.74)

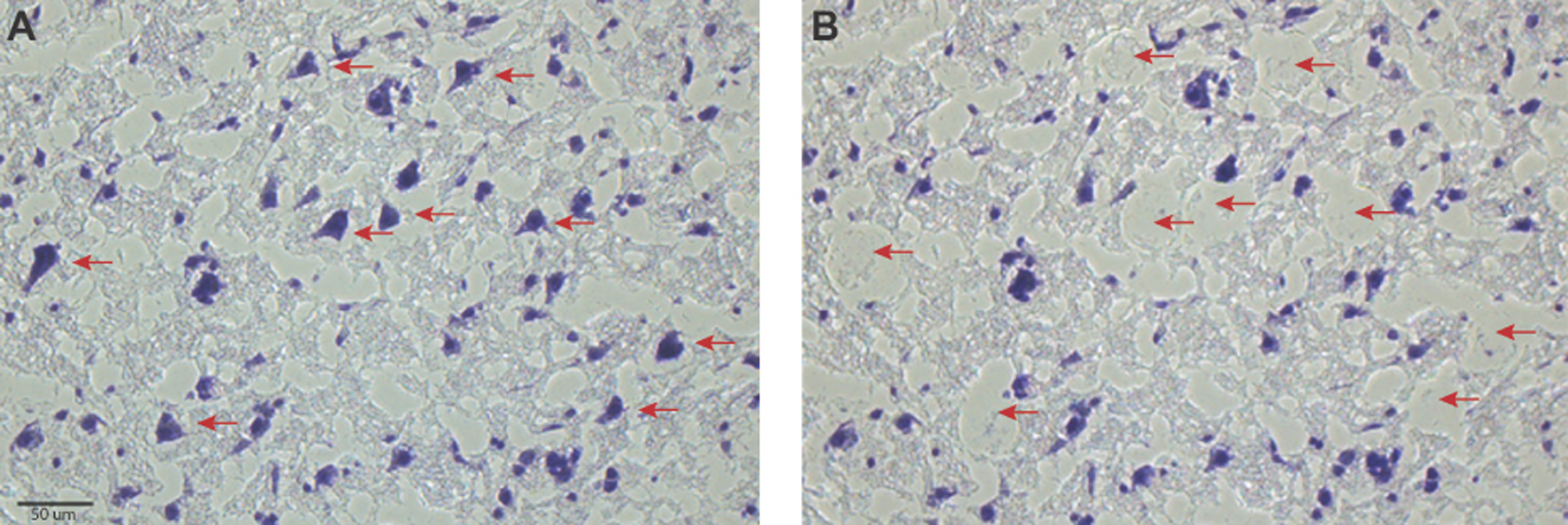

Supplement: Supplementary Figure 1 [file tp201574x1.tif]

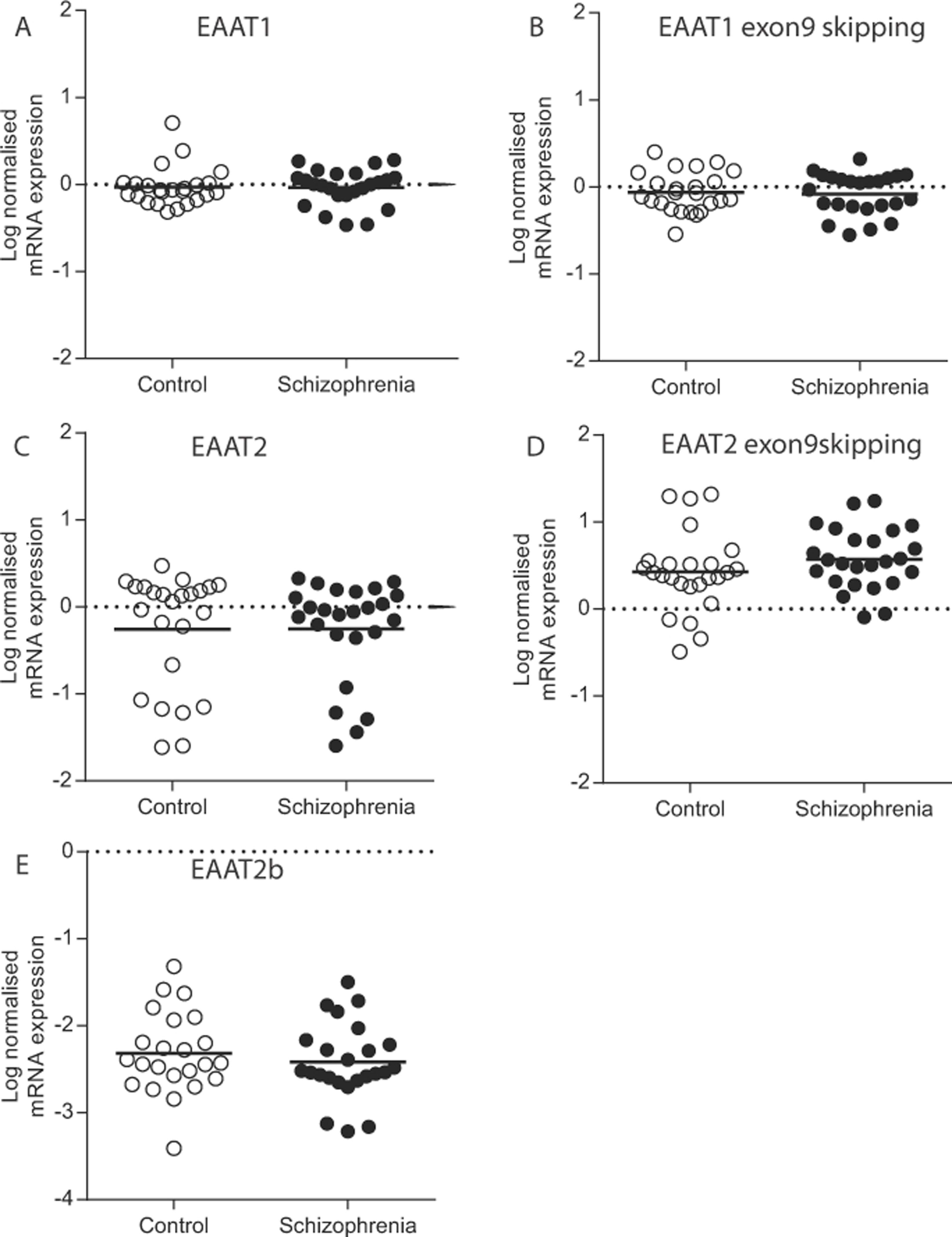

Supplement: Supplementary Figure 2 [file tp201574x2.tif]

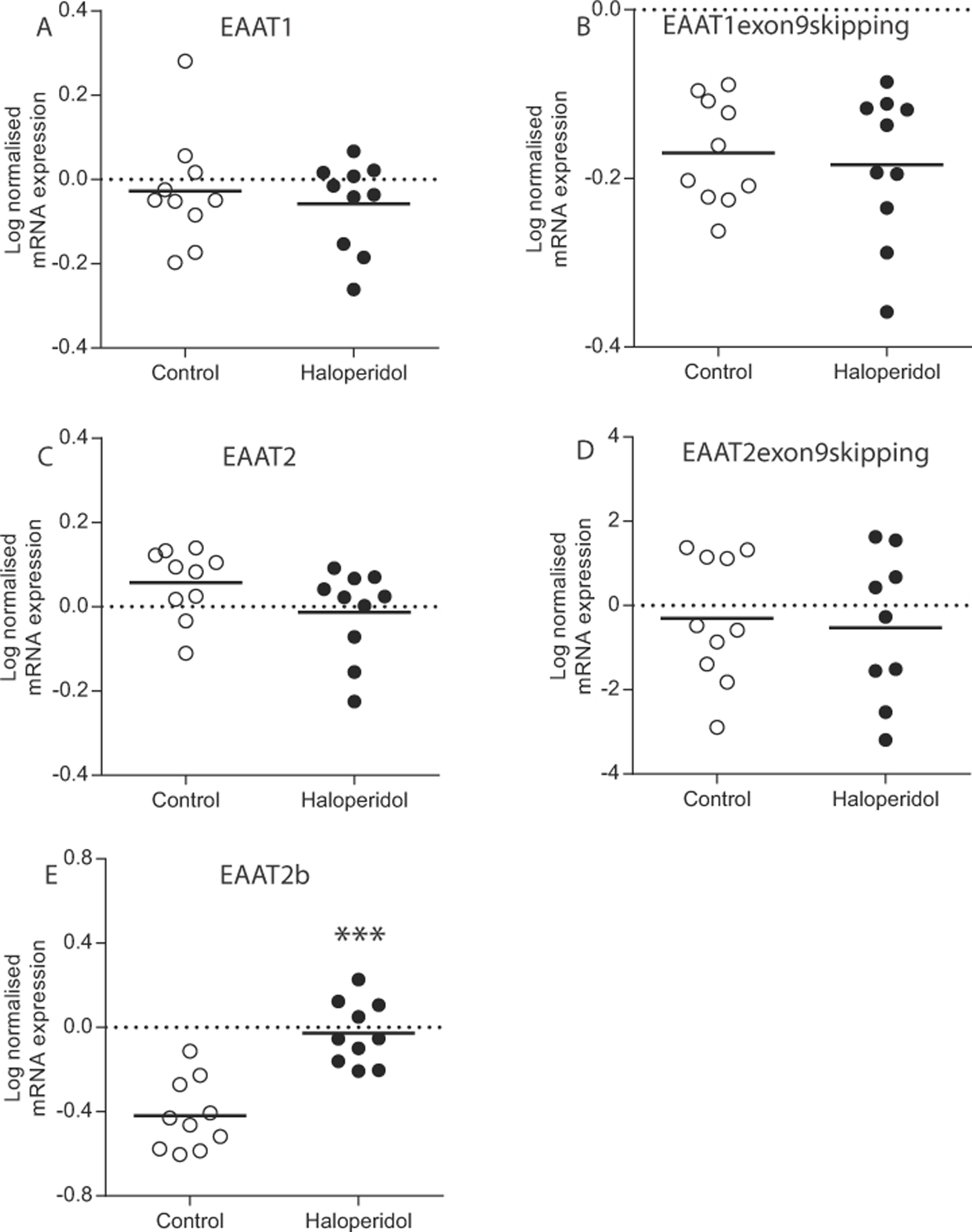

Supplement: Supplementary Figure 3 [file tp201574x3.tif]
