# Supplementary material for: Glutamate transporter splice variant expression in an enriched pyramidal cell population in schizophrenia
Source: Transl Psychiatry. 2015 Jun 9;5(6):e579–. doi: 10.1038/tp.2015.74 (PMC4490284; doi:10.1038/tp.2015.74)
Supplement: Supplementary Information [file tp201574x4.pdf]

Supplementary Table 1. Human SYBR-Green primer sequences

Primer	Forward	Reverse
**β-actin**	AGTACTCCGTGTGGATCGGC	GCTGATCCACATCTGCTGGA
**18S**	CGCCGCTAGAGGTGAAATTC	TTGGCAAATGCTTTCGCTC
**cyclophilinA**	CTCCTTTGAGCTGTTTGCAG	CACCACATGCTTGCCATCC
**GAPDH**	GACATGCCGCCTGGAGAAACC	GACAATGAATATGGCTACAGC
**EAAT1**	CGAAGCCATCATGAGACTGGTA	TCCCAGCAATCAGGAAGAGAA
**EAAT1exon9skipping**	TGCCCTCTATGAGGCTTTGG	GTCCGGAGGCGATCCCT
**EAAT2**	GGGCACCGCTTCCAGTG	ATACTGGCCGCGCCG
**EAAT2exon9skipping**	GGGCACCGCTTCCAGTG	GACTGAAGTTCTCATCCTGTCCCTT
**EAAT2b**	GGACAGGATGAGAACTTCAGTCAA	ACAAGTCTCGATATCCATGAATGG

Supplementary Table 2. Rat SYBR-Green primer sequences

Primer	Forward	Reverse
**β-actin**	AGTACTCTGTGTGGATCGGT	GCTGATCCACATCTGCTGGA
**18S**	CGCCGCTAGAGGTGAAATTC	TTGGCAAATGCTTTCGCTC
**cyclophilinA**	CTCCTTTGAGCTGTTTGCAG	CACCACATGCTTGCCATCC
**GAPDH**	GACATGCCGCCTGGAGAAACC	GACAATGAATATGGCTACAGC
**EAAT1**	TGACTGCCGCAGATGCCTTC	TGAGGAGGCCGACGATGACT
**EAAT1exon9skipping**	TGCCCTCTATGAGGCTTTGG	GTCCGGAGGCGATCCCT
**EAAT2**	CCAGTGCTGGAACTTTGCCT	TAAAGGGCTGTACCATCCAT
**EAAT2exon9skipping**	GCTGGGTATCGCCTGCTTGA	CATTCTATCCCTTACAGTCA
**EAAT2b**	GGACAGGATGAGAACTTCAGTCAA	ACAAGTCTCGATATCCATGAATGG

Supplementary Table 3 EAAT1 and EAAT2 allele frequencies and association with EAAT splice variants

**EAAT2 SNP**	**Allele**	**HWE**	**Frequency %**	**n**	**EAAT2**	**EAAT2ex9**	**EAAT2b**
rs7115246	A<br>G	0.05	54<br>46	17-19<br>26	**0.04***	**0.06(#)**	0.51
rs1570214	C<br>T	1	56<br>44	14-15<br>29-30	0.44	0.34	0.49
rs3818275	A<br>G	0.15	61<br>39	17-19<br>26	**0.04***	**0.002****	**0.01***
rs3794087	A<br>C	1	84<br>16	30-32<br>13	**0.05***	0.22	0.44
rs16927393	C<br>T	0.97	88<br>12	34-35<br>9-10	0.86	0.92	**0.03***
rs1885343	A<br>G	0.74	64<br>36	17-18<br>26-28	0.76	0.32	0.77
rs1923298	C<br>T	0.82	73<br>27	25-26<br>17-20	0.29	0.79	0.27
rs4756224	A<br>G	1	71<br>29	22-24<br>20-22	0.81	0.56	**0.06(#)**
rs4534557	C<br>G	0.8	60<br>40	15-18<br>25-27	0.26	0.75	0.68
rs4755404	C<br>G	0.83	61<br>39	17-18<br>26-27	0.11	**0.02***	0.24
rs1923294	A<br>G	1	54<br>59	13-14<br>29-30	0.57	0.38	0.50
**EAAT1 SNP**					1	**EAAT1ex9**	
rs2731880	C<br>T	0.87	55<br>45	13-14<br>30-32	0.95	0.50	
rs4869676	A<br>T	0.16	63<br>37	14-15<br>31-32	0.653	0.199	
rs1366632	G<br>T	0.42	50<br>50	13-14<br>33	0.29	0.28	
rs3776581	A<br>G	0.43	68<br>32	24<br>22-23	0.86	0.51	
rs1549627	C<br>T	0.83	51<br>49	13<br>33-34	0.96	0.93	
rs2269272	C<br>T	0.74	75<br>25	25-26<br>21	0.87	0.69	

The association between EAAT2 and EAAT1 polymorphisms and EAAT2 and EAAT1 splice variant mRNA expression are outlined in Supplementary Table 3. The table lists the SNP identifiers for EAAT2 and EAAT1 and the alleles and their observed % frequency. All subjects were in Hardy Weinberg equilibrium (HWE). The p-value resulting from Mann-

Whitney U analysis is listed. Results with a p-value <0.05 and results with a trend towards significance (p >0.05 but p<0.07) are highlighted **in bold**. Significant results are detailed in Figure 4. SNP, single nucleotide polymorphism; n, number of subjects; NA, not applicable; ns, not significant. *p<0.05, **p<0.01, # P<0.07.

Supplemental Figure legends

Supplementary Figure 1

Representative Nissl-stained pyramidal cells of the anterior cingulate cortex before (A) and after (B) capture by laser capture microdissection (LCM). Pyramidal cells targeted for capture are identified by arrows in (A). Locations of pyramidal cells captured by LCM are identified by arrows in (B).

Supplementary Figure 2

Region-level DLPFC log normalized mRNA expression of the EAAT splice variants EAAT1 (A), EAAT1 exon9skipping (B), EAAT2 (C), EAAT2 exon9skipping (D) and EAAT2b (E). Following Student's t-test analysis there was no significant difference in EAAT splice variant expression in control and schizophrenia subjects in the DLPFC region. Data expressed as mean ± SD, n=23-25 per group. DLPFC, dorsal lateral prefrontal cortex; EAAT, excitatory amino acid transporter.

Supplementary Figure 3

Rat haloperidol-treated region-level log normalized mRNA expression of the EAAT splice variants EAAT1 (A), EAAT1 exon9skipping (B), EAAT2 (C), EAAT2 exon9skipping (D) and EAAT2b (E). Following Student's t-test analysis there was a significant increase in EAAT2b splice variant expression in the frontal cortex of haloperidol treated animals compared to controls ($p<0.001$). Data expressed as mean ± SD, n=9-10 per group. ***$P<0.001$. EAAT, excitatory amino acid transporter.
